# Bilateral anophthalmia with septo-optic dysplasia

**DOI:** 10.4103/0974-620X.64233

**Published:** 2010

**Authors:** Manisha Jana, Sanjay Sharma

**Affiliations:** Department of Radiodiagnosis, All India Institute of Medical Sciences, New Delhi, India

**Keywords:** Anophthalmia, computed tomography, septo-optic dysplasia

## Abstract

Bilateral anophthalmia is a rare entity and association with septo-optic dysplasia is an even rare condition. The condition is characterized by absent eyeballs in the presence of eyelids, conjunctiva or lacrimal apparatus. Though anophthalmia can be diagnosed clinically, imaging plays a crucial role in delineating the associated anomalies. In addition, often clinical anophthalmia may prove to be severe microphthalmia on imaging. We describe the imaging findings in an infant with bilateral anophthalmia and septo-optic dysplasia.

## Introduction

Septo-optic dysplasia (de Morsier syndrome) is a rare entity described by Reeves in 1941 as a combination of optic nerve hypoplasia and absence of septum pellucidum.[1] Later on, an association with pituitary hypoplasia was described and septo-optic dysplasia is now often diagnosed clinically in presence of two or more features of the triad.[2] Anophthalmia refers to a complete absence of ocular tissue within the orbits. Microphthalmia is defined as the size of the eyeball at least two standard deviation less than the normal. In addition, often clinical anophthalmia may prove to be severe microphthalmia on imaging. The birth prevalence of anophthalmia is estimated to be three per 100,000[3] and the condition is commonly bilateral. Anophthalmia/ microphthalmia can either be isolated or syndromic and have complex etiology. When bilateral, anophthalmia has a high rate of associayed central nervous system abnormalities,[4] including septo-optic dysplasia, corpus callosum dysgenesis and pituitary anomalies.[5] Congenital anophthalmia associated with septo-optic dysplasia is a rare entity and not many cases are reported in English literature.[4,5]

## Case Report

**Figure 1:**
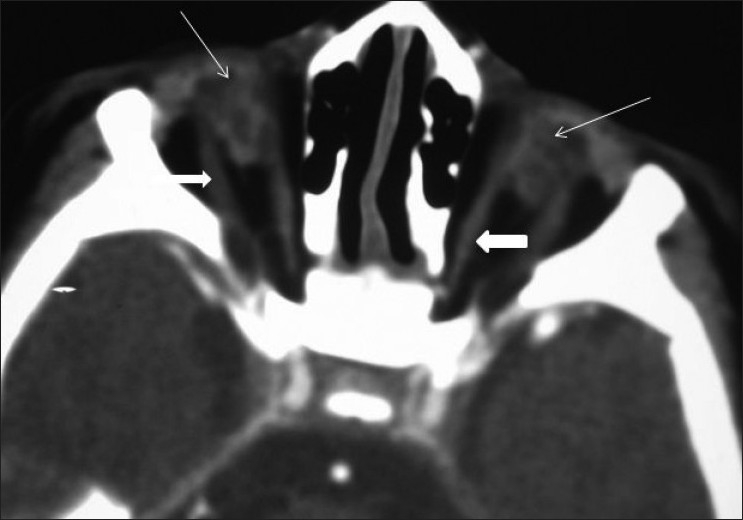
Axial CECT image of orbit showing disorganized soft tissue replacing bilateral eyeballs (arrows), absent bilateral optic nerves and narrow orbital diameter. The extra-ocular muscles are present bilaterally (block arrows)

**Figure 2:**
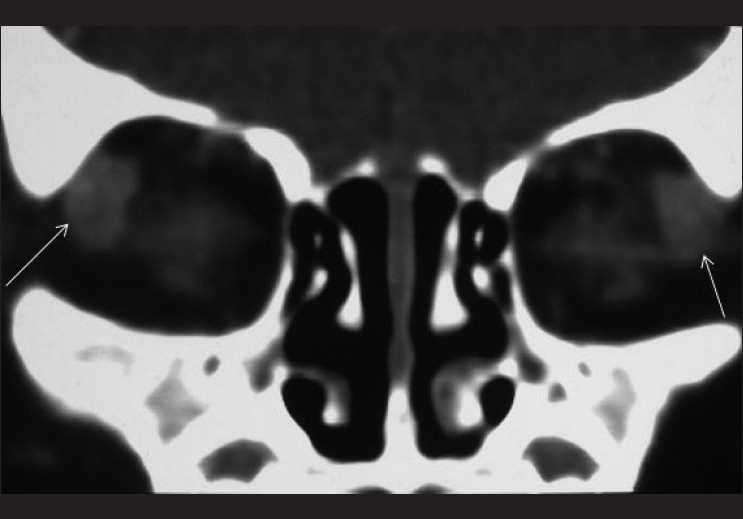
Coronal reformatted CECT image of the orbit shows absent eyeballs. The lacrimal glands are present bilaterally (arrows)

**Figure 3:**
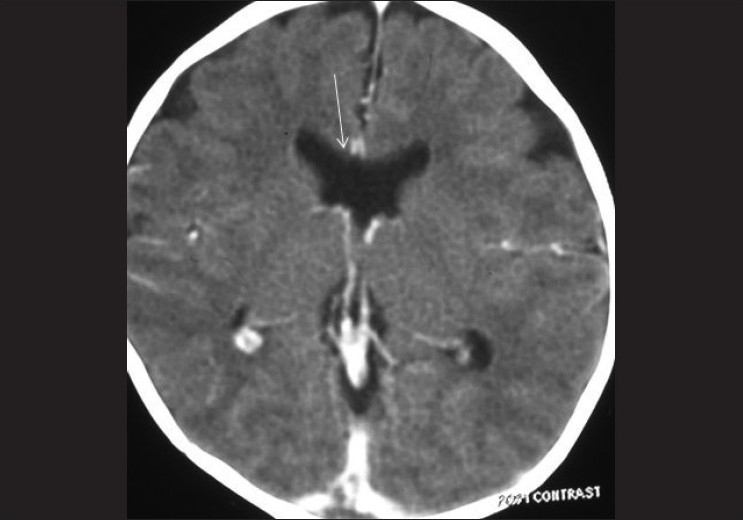
Axial CECT of brain reveals absence of the septum pellucidum (arrow) and squared configuration of the frontal horns of the lateral ventricles

A three-month-old female child presented with bilateral absence of eyeballs and delayed developmental milestones. At birth her milestones were normal. There was no history of congenital infections like toxoplasma or rubella or any significant teratogenic drug intake during pregnancy. There was no family history of anophthalmia/ microphthalmia or significant neurological anomalies. On examination, she had absent globes bilaterally. There was no associated dysmorphic facies orother neurological abnormalities. The auditory and motor system examinations were normal. Contrast-enhanced computed tomography (CT) of the orbit and brain was performed. It revealed bilateral absence of the globes (which were replaced by disorganized mass of soft tissue) and optic nerve. The orbital orbital diameter was reduced [Figure 1]. Ocular adnexae (the extraocular muscles, lid and lacrimal apparatus) were present [Figure 2]. CT of the brain revealed absence of septum pellucidum and squared configuration of bilateral frontal horns [Figure 3]. The pituitary gland was normal. She did not have any other associated anomalies. A diagnosis of septo-optic dysplasia with bilateral anophthalmia was made. She was taken up for bilateral, sequential orbital prosthesis implantation. Bilateral orbital prostheses implantation were performed at an interval of six months and the cosmetic outcome was good.

## Discussion

The development of the eyeball begins in the fourth week of gestation when optic grooves appear at the cranial end of the embryo.[6] Along with the development of the neural folds to form the forebrain, the optic grooves evaginate and form the optic vesicles. Subsequently, the distal ends of the vesicles grow and the connections with the forebrain constrict to form optic stalks (precursor of the optic nerves). The eyeballs are fully formed during fourth to eight weeks of gestation and the closure occurs along the embryonic fissure. Ganglion cell projections extend centrally through the optic stalk and form the optic nerves, which decussate to form the chiasm. Insults in early embryogenesis affecting the developing optic vesicle can result in anophthalmia (primary or secondary anophthalmia).[6] An insult in later embryogenesis results in degeneration of the visual elements already formed (degenerative anophthalmia).

Anophthalmia/ microphthalmia can be sporadic or syndromic. Several etiological factors have been implicated for example- consanguinity, maternal rubella infection, maternal vitamin A deficiency. Several syndromes including Triploidy syndrome, trisomy 13 (Patau syndrome), trisomy 18 (Edwards syndrome), Wolf-Hirschhorn syndrome and Several genetic mutations have been associated with anophthalmia/ microphthalmia syndromes.[7] Of all the chromosomal anomalies, only loss-of —function mutation of the SOX2 gene in chromosome 3 has been identified as a major causative factor of bilateral anophthalmia/microphthalmia.[8] The most common phenotypic presentation of this entity is bilateral anophthalmia; however, the ’SOX2 anophthalmia syndrome’ can involve multiple anomalies including persistent hyperplastic primary vitreous, optic disc dysplasia, cataracts, mental retardation, neurological abnormalities, facial dysmorphisma, esophageal pathologies, anomalies of the male genitalia.[9] Clinical studies found the SOX2 mutations to be associated with rare variant subtypes of septo-optic dysplasia, anterior pituitary hypoplasia and hypogonadotropic hypogonadism.[10]

Anophthalmia presents clinically as the absence of eyeballs. Associated neurological anomalies can manifest as mental retardation or developmental delay. Presentation of septo-optic dysplasia can be variable, depending on the CNS and endorinological anomalies. Imaging plays a crucial role in defining the ophthalmic and brain anomalies. In anophthalmia, the eyeball is completely absent and replaced by a disorganized soft tissue structure. Histologic examination has shown absence of neuroectodermal tissue.[11] On magnetic resonance imaging (MRI) the orbital soft tissue shows intermediate signal intensity on T1 weighted and low intensity on T2 weighted images. The ocular adnexae (the lid, conjunctiva, lacrimal glands) are present and orbital volume is reduced. Optic pathway and the extraocular muscles are variably present.[7] In microphthalmia the size of the eyeball is at least two standard deviation less than the normal population, with a smaller orbital size. However, the signal intensity of the lens and vitreous appears normal. Associated intracranial anomalies include absence of the anterior pituitary, agenesis of corps callosum, absence of septum pellucidum, dilatation of the ventricles, polymicrogyria. MRI is the ideal modality of imaging the visual pathway and should be done wherever feasible. The imaging protocol should be include T2weighted coronal images of the barin and the orbits for evaluation of the optic pathways and axial T1 and T2 weighted brain images for structural evaluation.

The management of anophthalmia is difficult. Anophthalmia can be treated with enucleation with implantationof prosthesis However, severe anophthalmia is often associated with orbital hypoplasia and poor outcome after prosthesis impantation. To conclude, bilateral anophthalmia is usually diagnosed clinically, but imaging plays a pivotal role in delineating the optic neural pathway and CNS anomalies, which have implications in prognosis.
